# Dysregulation of YAP by ARF Stimulated with Tea-derived Carbon Nanodots

**DOI:** 10.1038/s41598-017-16441-y

**Published:** 2017-11-29

**Authors:** Yingqiu Xie, Qinglei Sun, Ayan A. Nurkesh, Jiang Lu, Sholpan Kauanova, Jinhong Feng, Darkhan Tursynkhan, Qing Yang, Aishabibi Kassymbek, Mirat Karibayev, Korlan Duisenova, Haiyan Fan, Xiao Wang, Limara Manarbek, Aisulu Maipas, Zhenbang Chen, Mannix P. Balanay

**Affiliations:** 1 Department of Biology, School of Science and Technology, Nazarbayev University, Astana, 010000 Kazakhstan; 2 Shandong Analysis and Test Center, Shandong Academy of Sciences, 19 Keyuan Street, Jinan, 250014 China; 3 Department of Urology, Shenzhen University Luohu Hospital; Shenzhen Following Precision Medical Research Institute, Luohu Hospital Group, Shenzhen, 51800 China; 4 Department of Chemistry, School of Science and Technology, Nazarbayev University, Astana, 010000 Kazakhstan; 5 Department of Biochemistry and Cancer Biology, Meharry Medical College, Nashville, TN 37208 USA

## Abstract

YAP is a downstream nuclear transcription factor of Hippo pathway which plays an essential role in development, cell growth, organ size and homeostasis. It was previously identified that elevation of YAP in genomics of genetic engineered mouse (GEM) model of prostate cancer is associated with Pten/Trp53 inactivation and ARF elevation hypothesizing the essential crosstalk of AKT/mTOR/YAP with ARF in prostate cancer. However, the detailed function and trafficking of YAP in cancer cells remains unclear. Using GEM microarray model, we found ARF dysregulates Hippo and Wnt pathways. In particular, ARF knockdown reduced non-nuclear localization of YAP which led to an increase in F-actin. Mechanistically, ARF knockdown suppressed protein turnover of β-catenin/YAP, and therefore enhanced the activity of AKT and phosphorylation of YAP. Moreover, we found tea-derived carbon dots can interact with ARF in nucleus that may further lead to the non-nuclear localization of YAP. Thus, we reported a novel crosstalk of ARF/β-catenin dysregulated YAP in Hippo pathway and a new approach to stimulate ARF-mediated signaling to inhibit nuclear YAP using nanomaterials implicating an innovative avenue for treatment of cancer.

## Introduction

The yes-associated protein 1 (YAP) is a downstream effector of the Hippo pathway that is involved in the development and progress of numerous types of cancers. It is believed that YAP undergoes post-translational modification and cellular translocation which may localize in different cellular compartment such as cytosol, nucleus, membrane, and muscle fiber^1,2^. One of the key function of nuclear YAP is to regulate transcription of target genes involved in oncogenesis. Cytosolic YAP may function as a tumor suppressor through multiple signaling pathways such as inhibition of nuclear translocation of β-catenin and regulation of β-catenin degradation^3^. One study that focuses on the F-actin cytoskeleton shows that F-actin enhances YAP nuclear translocation^4^, It was found that the crosstalk between YAP and mTOR (mammalian target of rapamycin) is through Pten (phosphatase and tensin homolog)/AKT (serine/threonine kinase)^5^. This feedback loop suggests the reversible regulation of YAP by mTOR^6^. Thereby targeting YAP by rapamycin might disrupt the cytoskeleton to inhibit cancer cell migration. In addition, AKT can directly phosphorylate YAP at S127 for regulation of binding to 14-3-3 in cytosol and nuclear translocation^7,8^. Thus, inhibition of AKT/mTOR signaling may suppress the oncogenic function of nuclear YAP through regulation of nuclear-cytosol shuttling. However, the detailed function and signaling pathways of YAP in non-nuclear translocation such as membrane compartment and cytosol remains unclear even it is found that YAP is localized at the membrane^2^.

ARF (alternative reading frame protein product of the cyclin-dependent kinase inhibitor 2A (CDKN2A) locus, p14^ARF^ in human and p19^ARF^ in mouse) is originally identified at ARF-INK4a locus on chromosome 9q21 in humans^9^. The classical ARF pathway functions as a tumor suppressive mechanism through coupling with p53 protein to induce cellular senescence, inhibit ribosomal RNA transcription and processing, or activate autophagy^10,11^. We previously demonstrated that ARF stabilizes SLUG to promote epithelial-mesenchymal transition (EMT) in prostate cancer (PCa) *in vitro* and *in vivo* through degradation of cell adhesion^12^. Moreover, ARF regulates tumor microenvironment through MMP7 (matrix metalloproteinase-7) nuclear translocation^13^. Most importantly, it was found that Pten/Trp53 loss induces co-elevation of ARF and YAP^14,15^, which might be a crosstalk between the two pathways. We utilized the Pten/Trp53 null mouse model of cancer and bioinformatics approaches to investigate the noncanonical ARF signaling and its regulation in cancer.

Carbon dots (C-dots) have emerged as a promising nanoparticle for various applications such as drug delivery and anti-cancer agents having an average size below 10 nm with interesting properties such as high photostability, low cost, and impressive biocompatibility^16–21^. Although it was commonly believed that C-dots possess low cytotoxicity, the inhibition against cancer cell even at low dose was observed in the C-dots developed from tea and ginger^22,23^. In the present paper, we attempted to combine C-dots with rapamycin to explore synergic effects and at the same time obtaining some mechanistic insights. Our results revealed a genetic landscape mediated by p19^Arf^ in prostate tumors *in vivo* and further identified a novel ARF/β-catenin/YAP signaling pathway that regulates YAP nuclear translocation. Targeting these signaling through C-dots suggests a novel avenue for treatment of cancer.

## Results

### Regulation of Hippo and Wnt pathways based on genetic engineered mouse model of p19^Arf^

**Figure 1:**
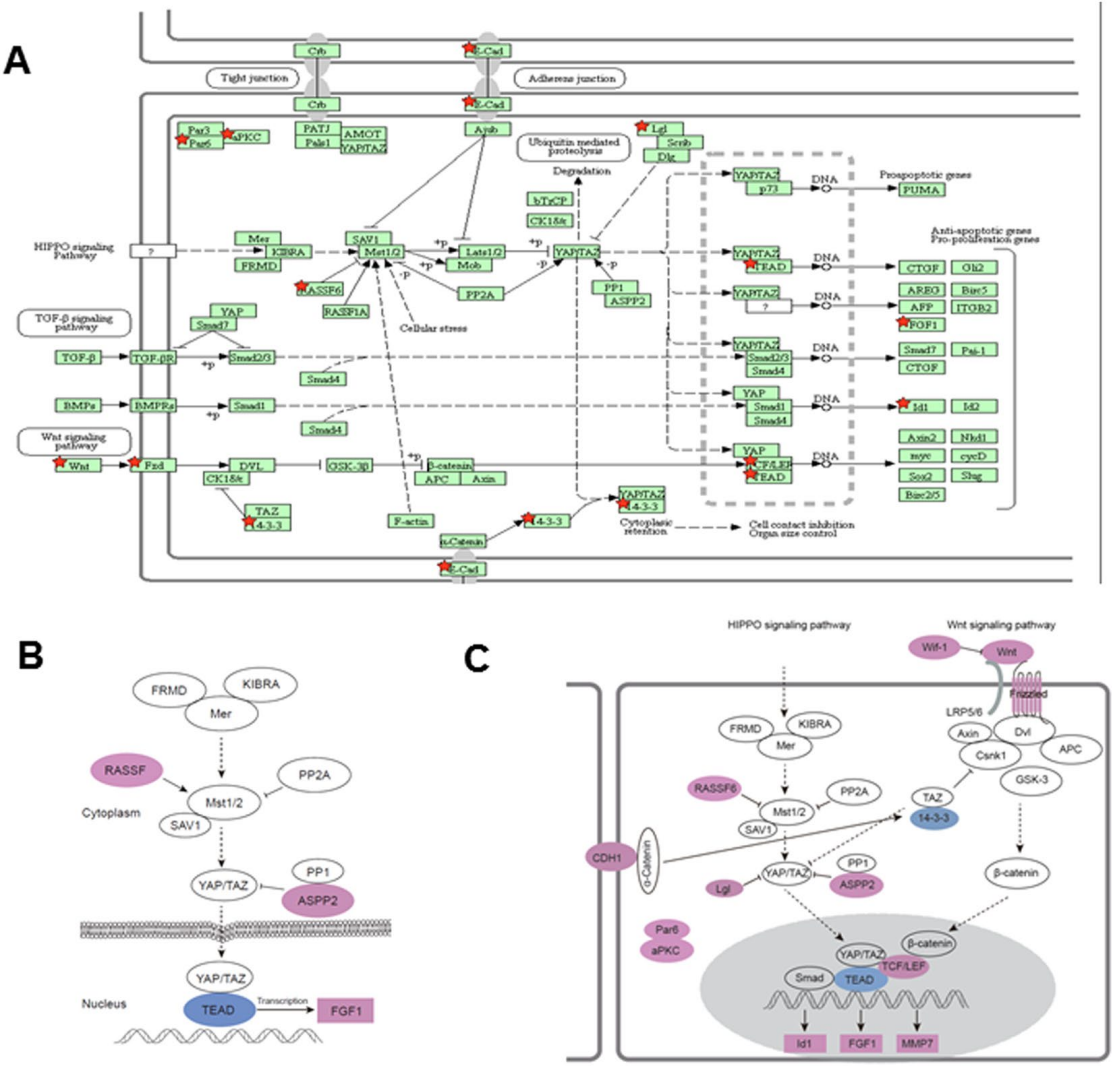
ARF dysregulates multiple signaling pathways *in vivo* in genetic engineering mouse model of prostate cancer. Pten/Trp53 knockout mice were used for further knockout of p19^Arf(+/–)^. The prostate tissue samples were collected for microarray analysis. Gene expression profiles were compared within the two groups and re-analyzed for pathways using DAVID online tools. **(A)** Pathways including Hippo and Wnt were found in the whole profiles analysis. The differential expressed genes were marked as red color of star shape. **(B)** and **(C)** Gene expression of Hippo and Wnt pathways^24,25^, respectively. The blue color indicates the downregulation and pink color indicates the upregulation.

Previously, a *Pten/Trp53/p19* ^*Arf*^ triple knockout mouse model was applied to study the function of p19^Arf^ in the context of *Pten/Trp53* mutation and found that there is micro-environmental regulation by ARF upon oncogenic stress of mutation of Pten and p53^13^. We further re-analyzed the gene expression profiles upon p19^Arf^ knockout in mice using DAVID online tool for pathways^24,25^. Compared to *Pten/Trp53* mice, *Pten/Trp53/p19* ^*Arf*^ triple knockout mice exhibited different pathways in gene expressions. We found 13 genes (*Rassf6, Cdh1, Ywhag, Tead1, Fgf1, Fzd3, Id1, Llgl2, Pard6b, Prkcz, Tcf7l2, Trp53bp2, Wnt4*) were classed into Hippo signaling pathway, and 7 genes (*Wif1, Camk2b, Fzd3, Mmp7, Prkcb, Tcf7l2, Wnt4*) with Wnt signaling pathway (Fig. 1A). Given the crosstalk between Wnt and Hippo pathways for regulation of YAP associated cytoskeleton^26^, in a combined analysis of pathways, ARF likely regulates the crosstalk between YAP and Wnt pathway through 14-3-3 node in cytosol and β-catenin node in nucleus (Fig. 1B and C).

### Non-nuclear localization and function of YAP regulated by ARF

An investigation of the role of ARF in regulation of YAP shows that there is an abnormal regulation of ARF and Hippo pathway target protein YAP in prostate cancer^27,28^. In our previous studies of prostate cancer PC3 cells^12^, ARF knockdown inhibited cell growth, migration and decreased EMT. Using similar shRNA knockdown approach described previously, we found that ARF knockdown reduced non-nuclear localization of YAP and YAP nuclear localization became dominated by ARF knockdown concurrently (Fig. 1 and Supplementary Fig. 1). Thus, ARF may enhance the expression levels of cytosolic and membrane localized YAP. Our data suggests that ARF is essential in the regulation of non-nuclear YAP pathway.

**Figure 2:**
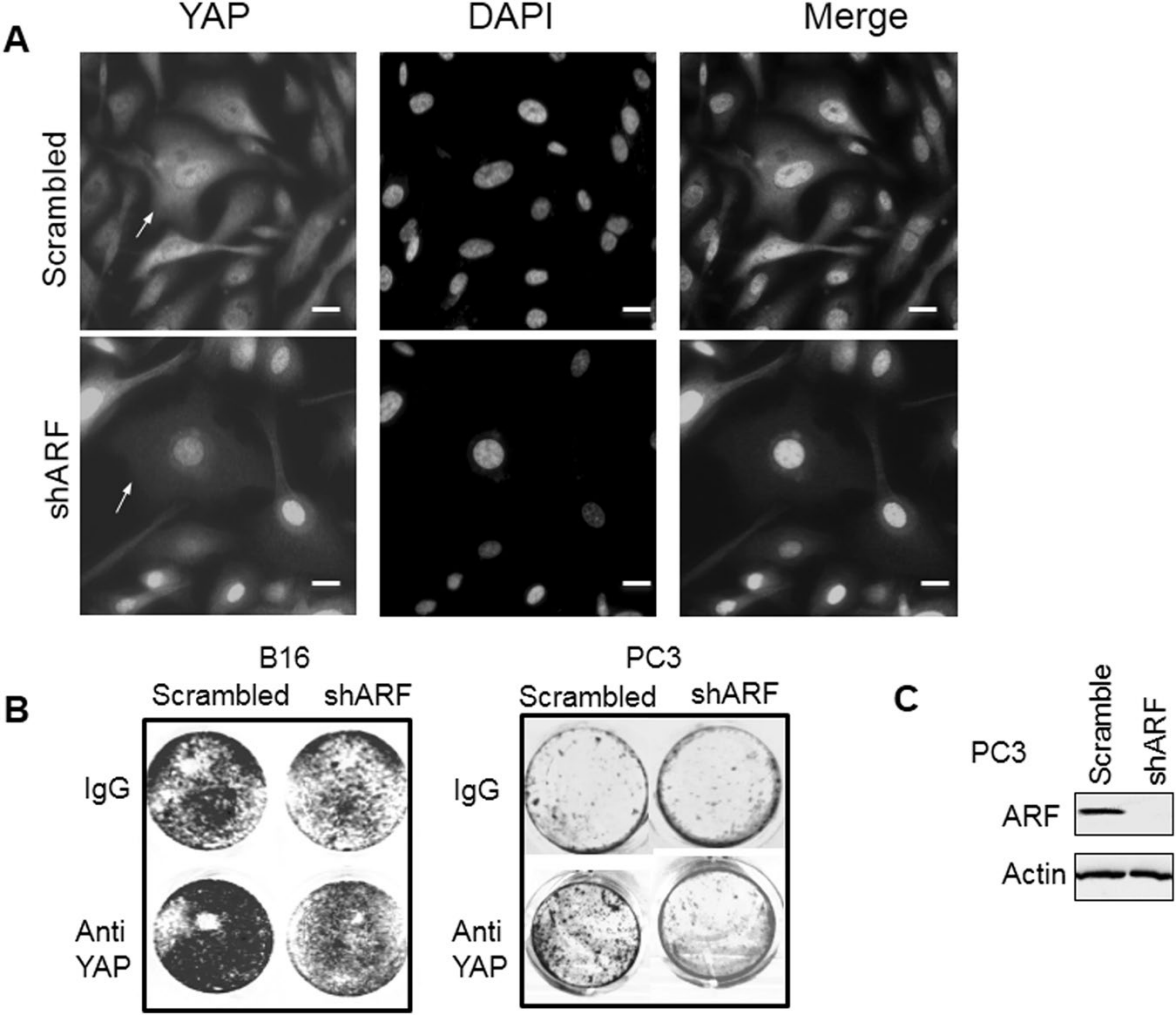
Dysregulated YAP in cytosol by ARF. **(A)** ARF knockdown decreases non-nuclear YAP in PC3 cells. **(B)** Treatment of cancer cells by Anti-YAP antibody increases cell growth in an ARF-dependent manner. **(C)** Western blot showing ARF knockdown by shRNA in PC3 cells. Scale bars, 20 µm.

The role of ARF mediated changes in cellular compartmental YAP was investigated given that the tumor suppressive function of non-nuclear YAP by testing the combined effect of ARF knockdown and YAP depletion. As shown in Fig. 2, the ARF knockdown suppresses cell growth of human prostate PC3 and mouse melanoma B16 cells which is consistent with our previous results^12^. Upon depletion of predominant membrane YAP alone, cell growth was elevated. However, upon knockdown of ARF, membrane YAP depletion induced cell growth elevation was disputed. Thus, our data suggest that tumor suppressive-like function of membrane YAP requires ARF.

### Stability and cellular localization of YAP by ARF

**Figure 3:**
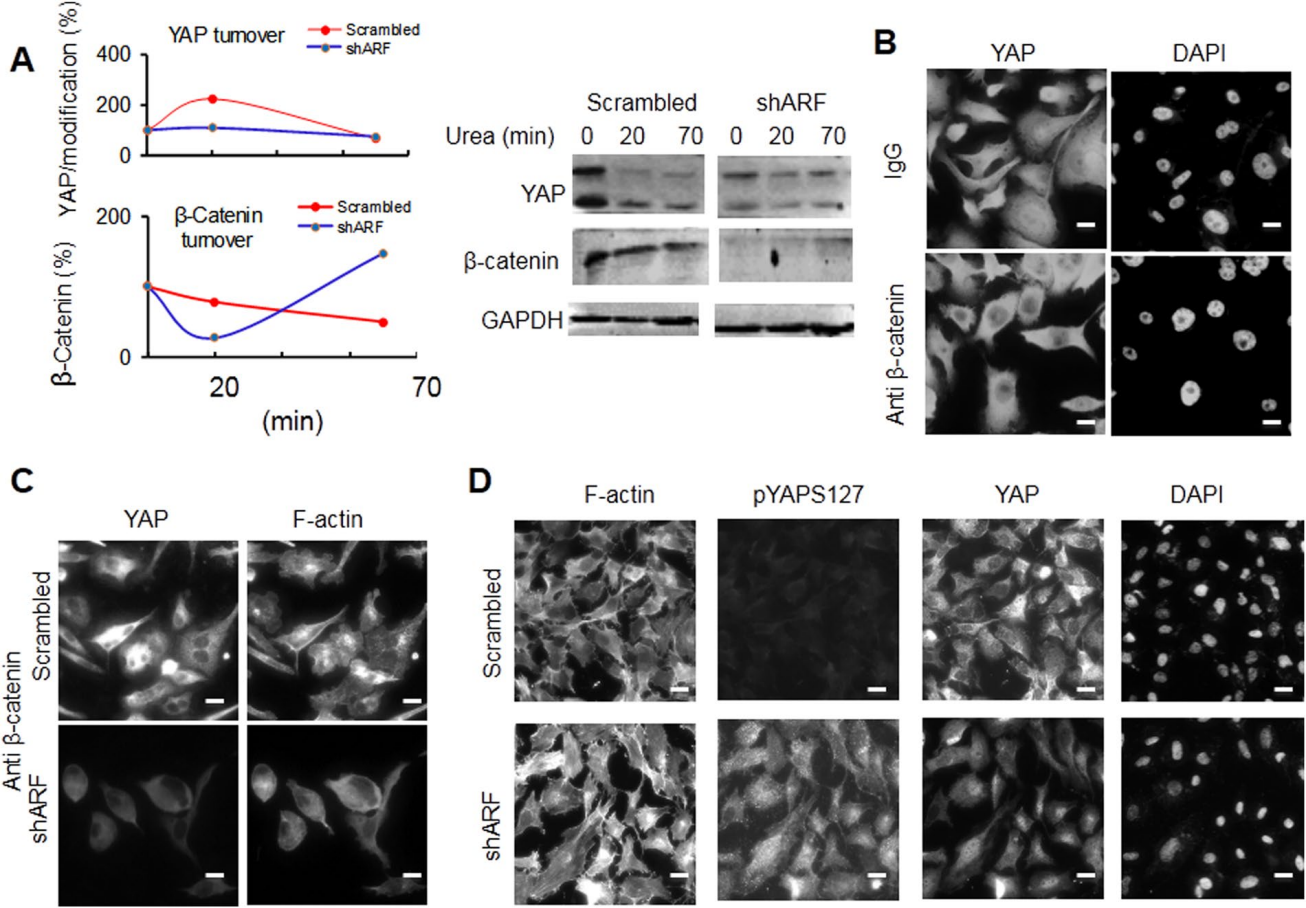
ARF dysregulated stability, nuclear localization of YAP and associated F-actin formation by crosstalk with β-catenin. (**A**) ARF knockdown decreases folding of YAP in PC3 cells. **(B)** and **(C)** Treatment of PC3 cells by Anti-β-catenin antibody decreases nuclear YAP and F-actin, respectively in an ARF-dependent manner. **(D)** ARF knockdown increases phosphorylation of YAP at S127 (pYAPS127) in PC3 cells. Scale bars, 20 µm.

To explore the mechanism of ARF mediated YAP regulation, we determine the effect of ARF knockdown on stability of YAP as well as YAP regulator β-catenin. As presented in Fig. 3, ARF knockdown reduced expression levels of YAP and its tumor suppressive regulator β-catenin. Upon treatment of cells with low dose of urea, the protein undergoes reversible denaturation and degradation due to un-stabilized structures. ARF knockdown suppressed the reversible process of protein denaturation of YAP. Moreover, ARF knockdown decelerated protein turnover of β-catenin. This could be inferred that the ARF regulates β-catenin/YAP stability through protein turnover.

Given the essential role of β-catenin in regulation of YAP, we tested further the effect of depletion of β-catenin using antibodies on YAP nuclear translocation in PC3 cells. We found that β-catenin is indeed critical for nuclear localization of YAP. The combined shARF and anti-β-catenin not only reduced nuclear localization of YAP but also F-actin formation. In addition, the knockdown of ARF, which enhanced phosphorylation of YAP at S127 (Fig. 3D and Supplementary Fig. 1), is associated with dominant nuclear localization of YAP and increased F-actin. Given that AKT is one of the kinases phosphorylating YAP at S127, our results suggest that ARF may dysregulate YAP and associated cytoskeletal changes through phosphorylation of YAP possibly by AKT.

### Effect of ARF overexpression

**Figure 4:**
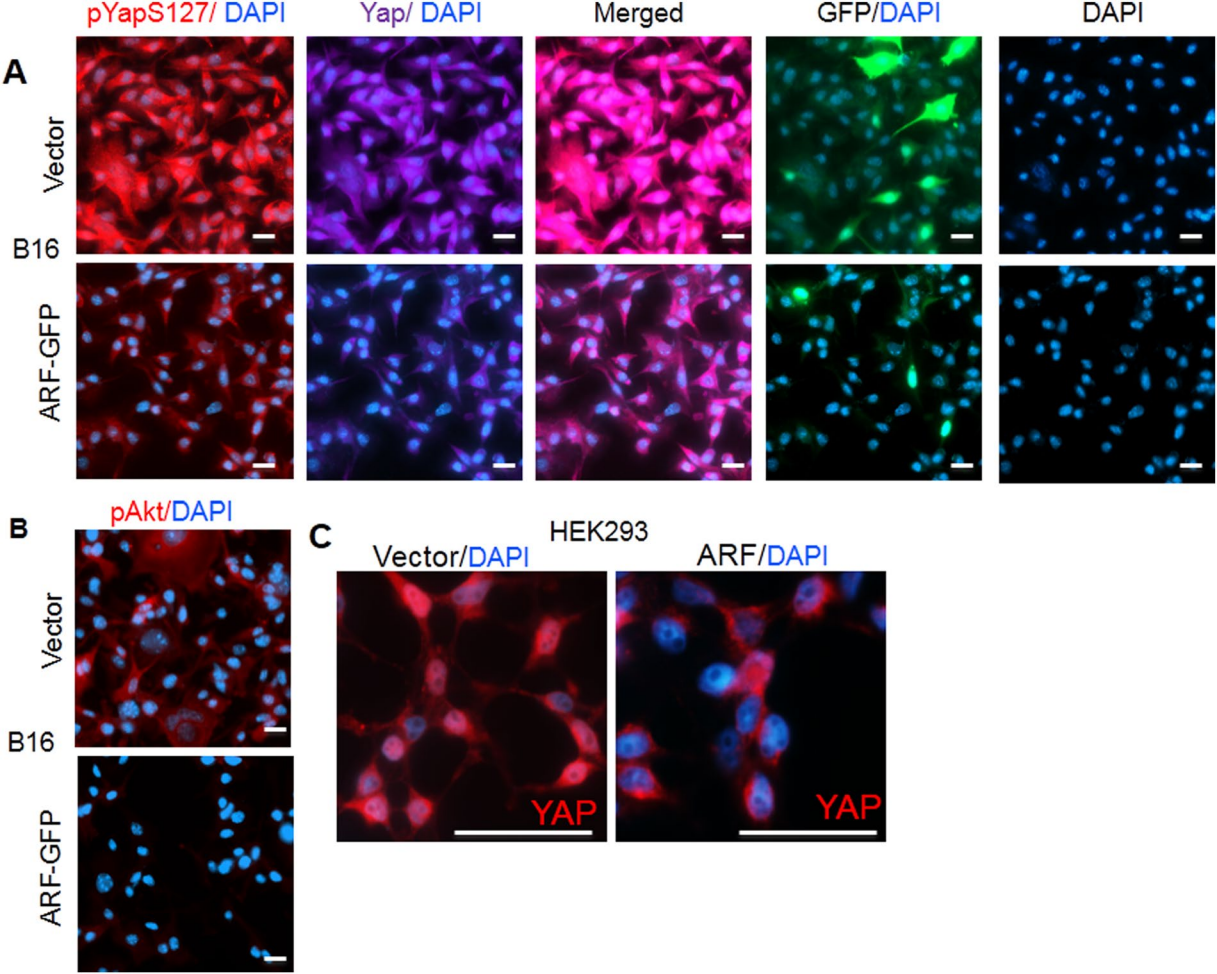
Dysregulated phosphorylation, nuclear localization of YAP and associated F-actin formation by ARF-mediated crosstalk with AKT. (**A**) and (**B**) ARF overexpression decreases phosphorylation of YAP at S127, pAKT and nuclear YAP in B16 cells, respectively. (**C**) ARF overexpression increases cytosolic but decreases nuclear YAP in HEK293 cells. Scale bars, 20 µm.

B16 and HEK293 cells which express very low levels of ARF (or Arf) were utilized to confirm whether overexpression of ARF can dysregulate YAP in nucleus. Our data shows that the overexpression of ARF-GFP fusion protein has reduced the phosphorylation of YAP at S127, and nucleus localization of YAP (Fig. 4). Consistently, we found that AKT activity measured by pAKT (S473) antibody decreased upon ARF elevation in B16 cells. In HEK293 cells which express moderate levels of YAP in nucleus, ARF overexpression also led to a decrease in YAP nucleus localization. These suggest that ARF sustains non-nuclear YAP.

### Influence of Rapamycin in β-catenin and YAP nuclear localization

**Figure 5:**
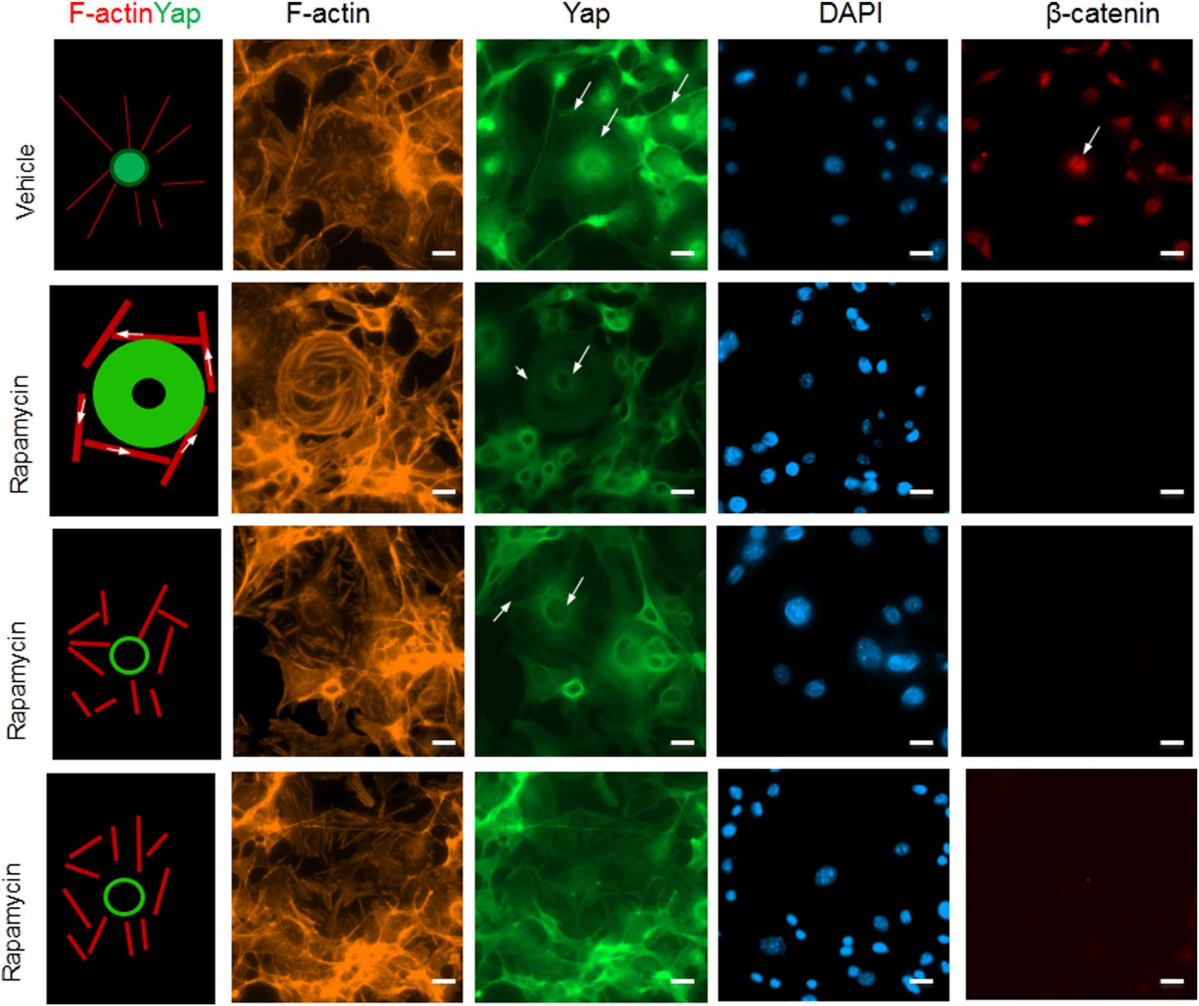
Dysregulated nuclear localization of YAP and associated F-actin formation by Rapamycin. B16 cells were serum starved for 1 hour then treated with serum and DMSO control or 0.1 µM Rapamycin for 10 min. Cells then fixed and subjected to IF analysis using confocal microscopy. Scale bars, 20 µm.

Since the AKT/mTOR/YAP feedback loop is mostly responsible for the regulation of YAP, we want to determine if the inhibition of mTOR by rapamycin can either repress nuclear localization of YAP or it is associated with the dysregulation of F-actin. For this, we utilized B16 cells since it showed enlarged F-actin. As observed in Fig. 5, we found rapamycin reduced YAP nuclear localization associated with changes in F-actin. The nuclear β-catenin was also reduced upon rapamycin treatment. Most importantly, rapamycin induced YAP translocation from nucleus to nuclear region. This type of localization of YAP associated with F-actin reorganization from radiation style to clockwise rotation style. This implies that the decrease in YAP nuclear localization caused by rapamycin may be through F-actin mediated dysregulation of activity of β-catenin.

### Characterization of tea-derived C-dot and binding in amino acids

**Figure 6:**
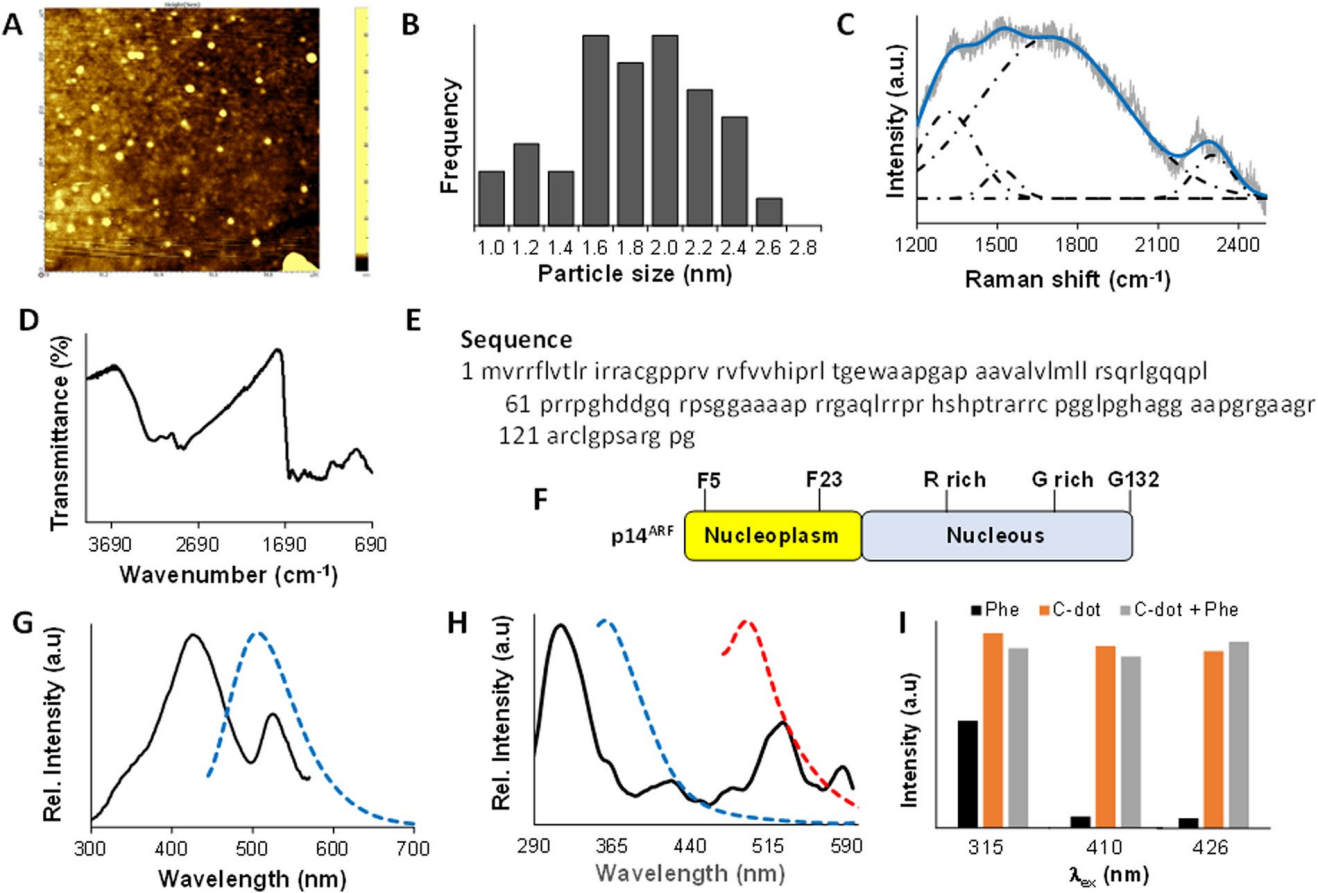
Characterization of tea-derived C-dot and binding in amino acids. **(A)** AFM image of the tea-derived C-dot deposited on a glass substrate. **(B)** Size distribution histogram of the C-dot. **(C)** Raman spectrum of the C-dot with peak deconvolution. **(D)** ATR FT-IR spectrum of the C-dot. **(E)** Amino acid sequence of the full length p14^ARF^ (AA: 2–132). **(F)** Schematic diagram of the N-terminal region of p14^ARF^ with phenylalanine. **(G)** Fluorescence excitation (black solid line) and emission (blue dotted line) of the C-dot. **(H)** Fluorescence excitation (black solid line) and emission (blue dotted line, λ_ex_ = 315 nm and red dotted line, λ_ex_ = 410 nm) of phenylalanine. **(I)** A comparison of the fluorescence intensity of phenylalanine (black), C-dot (orange), and C-dot + phenylalanine (gray).

Atomic force microscopy result suggested that the synthesized carbon dots have an average size of 1.8 ± 0.2 nm (Fig. 6A and B). The particles that show the size greater the 3 nm were believed in a form of aggregation. The Raman spectra of the C-dot excited at 633 nm is shown in Fig. 6C which shows four characteristic bands based on peak deconvolution using OriginPro 9.1 in the range of 1200–2500 cm^−1^. These four bands are likely coming from the graphene structure, wherein the first two bands at 1318 and 1516 cm^−1^ represent the D and G bands, respectively of the graphene sheet. The D band is the result of the structure defects due to the breathing modes of the six-atom ring, while the G band is coming from the *sp* ^2^ hybridized carbon^29,30^. The other two Raman shifts at 1701 and 2301 cm^−1^ are mostly likely from the stacking pattern of the graphene layers^31^. The appearance of 2301 cm^−1^, which are not commonly present in graphene samples but was observed in single-walled carbon nanotubes^32^, was coming from the thin-layer stacking of aggregated C-dots. The FT-IR spectra of the C-dot is presented in Fig. 6D, where it shows the C–H (2863 and 2925 cm^−1^) and C=C (1490 and 1549 cm^−1^) stretching vibrations representing the graphene layer. The peak at 1647 cm^−1^ along with the peaks at 3061 and 3224 cm^−1^ supports a functional group like the primary amide, where the first peak corresponds to the *v* (C=O) vibration in amide, and the latter two peaks represent N-H stretching. While the IR peaks at 1020 and 1420 cm^−1^ could be assigned to the C–N vibrations. These further supports the presence of amide bonds on the C-dot surface which was also observed on previously synthesized nitrogen-doped C-dots^33–35^.

In the previous binding assay of aminoacyl tRNA synthetase complex-interacting multifunctional protein 2 splice variant which was induced by the oncogenes in human lung cancer tissues and cells, it was found that it binds to the N-terminal region of p14^ARF^ (AA-2-29, Fig. 6E)^36^. At the nucleoplasm part of this region, contains some phenylalanine amino acids which would likely be responsible for its binding (Fig. 6F). Thus, we studied the binding between C-dots and phenylalanine by observing the fluorescence activity. The fluorescence excitation spectrum of the C-dot shows two distinct peaks centered at 426 and 523 nm and the corresponding emission spectrum for λ_ex_ = 426 nm are presented in Fig. 6G. Nearly no fluorescence was observed at the excitation wavelength of 523 nm. On the other hand, the fluorescence excitation spectrum of the phenylalanine (F) exhibits peaks at 315, 410, and 530 nm and the corresponding emission spectrum for the first two excitations are given in Fig. 6H. Figure 6I shows the fluorescence activity of the phenylalanine, C-dot, and the combination of C-dot and phenylalanine at excitation wavelengths of 315, 410, and 426 nm respectively. The first two are taken from the excitation wavelengths of the phenylalanine, while the last excitation energy was from the C-dot. Based on Fig. 6I, the fluorescence of C-dots at the excitation wavelength of 315 nm and 410 nm was quenched with the combination of phenylalanine, while the fluorescence of C-dots at the excitation wavelength of 426 nm was enhanced with the combination of phenylalanine. These results could indicate a significant binding between the C-dot and phenylalanine.

### Effect of tea-derived C-dots on ARF, dysregulation of YAP/F-actin signaling, cell growth and migration

**Figure 7:**
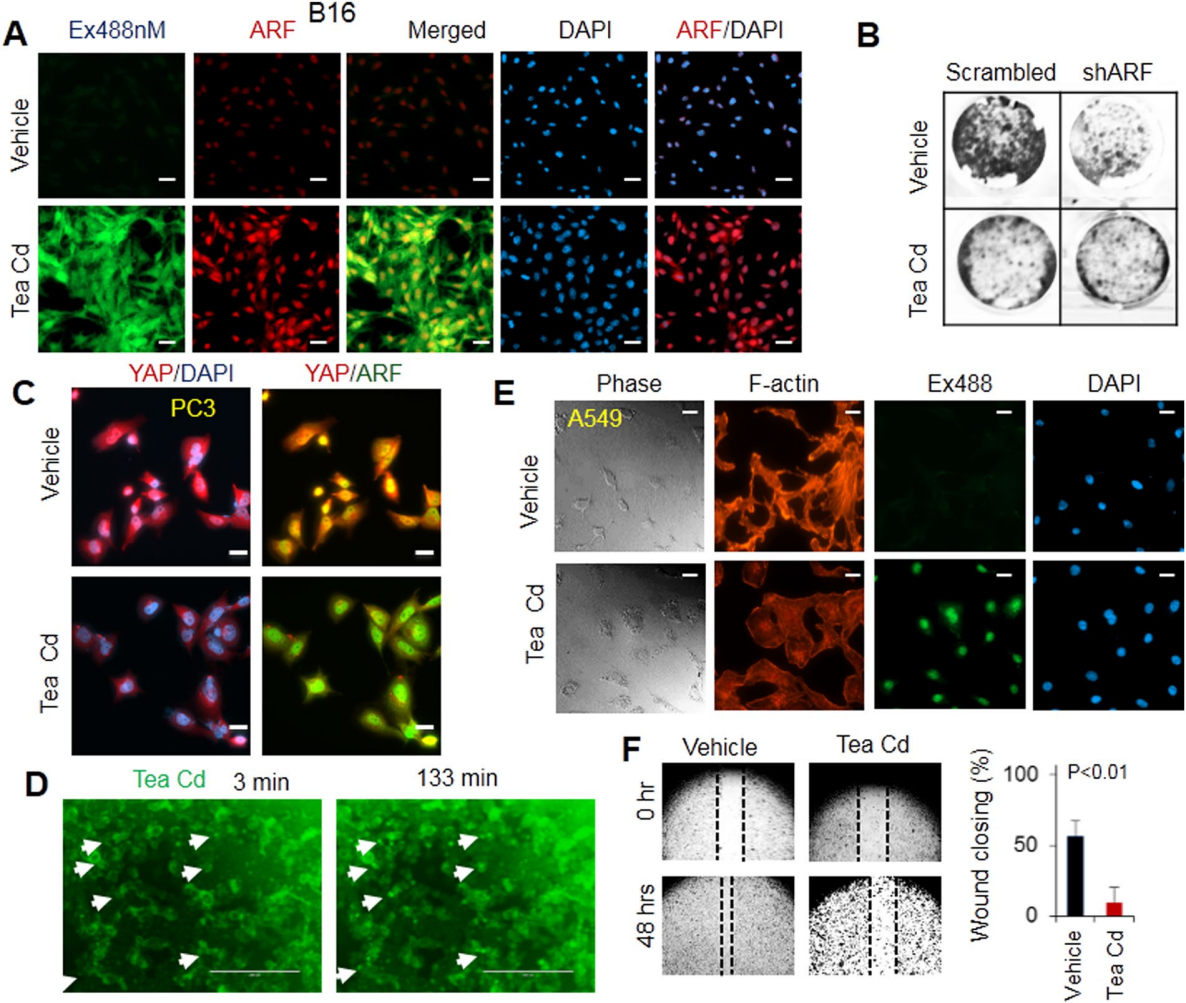
Dysregulated phosphorylation, nuclear localization of YAP and associated F-actin formation by C-dots which stimulate ARF. **(A)** C-dots increases ARF expression at 0.2 mg/ml in PC3 cells. **(B)** 0.1 mg/ml C-dots decreases cell growth in an ARF-dependent manner in PC3 cells. **(C)** 0.2 mg/ml C-dots decreases YAP nuclear localization and the interactions between ARF and YAP in PC3 cells. **(D)** Time lapse of C-dots localization in nucleus in MCF7 cells. **(E)** 0.2 mg/ml C-dots decreases F-actin in lung cancer A549 cells. **(F)** 0.2 mg/ml C-dots decreases cell migration in PC3 cells. Scale bars, 20 µm.

It was shown that C-dots derived from ginger can give cells stress by activating p53 pathways to inhibit cancer cell growth^23^. Given that ARF is upstream inducer of p53, we tested whether C-dots derived from tea can also induce cell stress to stimulate ARF expression. As presented in our results, the ARF expression was elevated in B16 cells upon treatment of as-prepared C-dots at 0.2 mg/ml for 3 hours (Fig. 7A). The fact that we observed ARF and C-dots were co-localized in nucleus convinces the potential interaction between C-dots and ARF (Fig. 7B and C).

Treatment of PC3 cells with the combination of C-dots and shRNA of ARF was performed to identify the essential role of ARF pathway in the C-dots mediated inhibition of cell growth. As presented in Fig. 7B, the C-dots alone inhibited cell growth, however, the inhibition effect is minimum upon ARF knockdown. Even though ARF induces cell death through multiple pathways including p53-dependent and -independent pathways, it is possible that ARF is the mediator for the tea-derived C-dots induced cell death. Our current study on the correlation between C-dots and the DNA damage and cell death is expected to reveal a more detailed mechanism for the C-dot induced inhibition of cancer cell growth. The reduction of cell growth or limited migration at lower dose of C-dots can may be due to multiple signaling pathways including ARF/YAP as YAP activation that lead to the inhibition of cancer cells^37^. Since Hippo activation induces cell death, the C-dots targeting ARF/YAP may thereby induce cell death.

The dynamic cellular translocation of C-dots in cells was carried out by treating B16 cells with C-dots at 0.2 mg/ml during different period of times and take multiple images by time-lapse microscopy (Fig. 7D). It was found at early stages, most cells uptake C-dots in cytosol and membrane. After more than 2 hours the C-dots was observed to enter into the nucleus. Additional tests were done to identify whether C-dots may inhibit YAP nuclear localization and can be associated with the decrease of F-actin as presented in Fig. 7E. The C-dots treated A549 cells have shown less actin stress fiber compared to vehicle control upon 24 hours post-treatment. This suggests that C-dots may disrupt F-actin formation through ARF/YAP. Moreover, the changes in F-actin were induced by C-dots and the PC3 cell migration is abrogated by treatment at a dose of 0.2 mg/ml (Fig. 7F).

**Figure 8:**
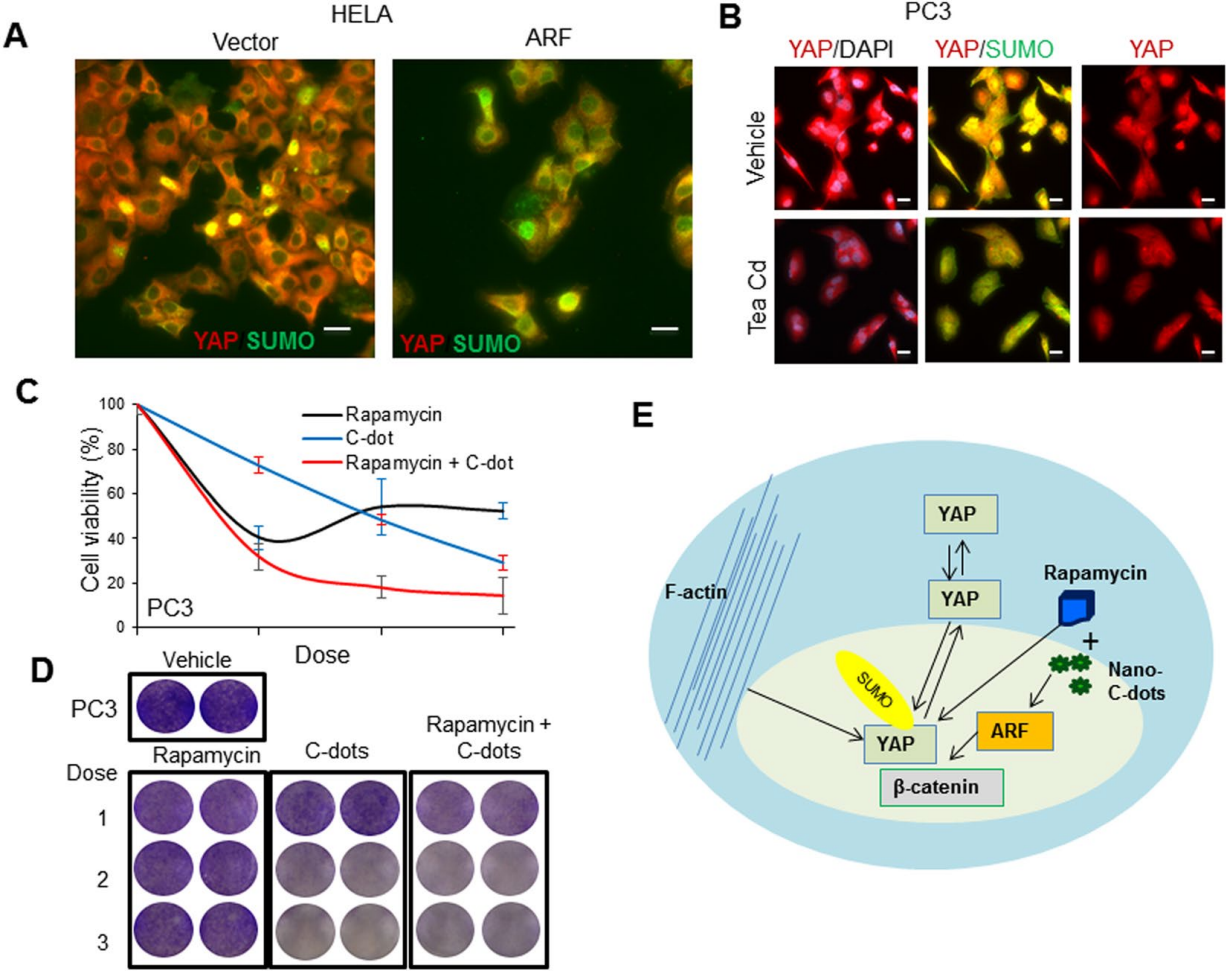
Tea-derived carbon nanodots dysregulate SUMOylation and sensitize cancer cells to Rapamycin. **(A)** ARF overexpression increases co-localization of SUMO and non-nuclear YAP in HELA cells. **(B)** Treatment of cancer cells by C-dots increases co-localization of SUMO and non-nuclear YAP in PC3 cells. (**C**) and **(D)** Treatment of cancer cells by C-dots sensitizes PC3 cells to rapamycin. **(E)** A proposed ARF/YAP regulatory model and inhibition of YAP by combinatorial targeting. Scale bars, 20 µm.

It was shown that ARF regulates protein SUMOylation which may regulate protein trafficking or shuttling between cytosol and nucleus^38^. An investigation of the post-translational modification of YAP by IF analysis of the interaction between YAP and SUMO was done to explore the mechanism of ARF and C-dots induced changes in non-nuclear YAP as shown in Fig. 8A. The data shows that the ARF overexpression induced the co-localization of YAP and SUMO in cytosol but not in nucleus thereby dysregulating overall SUMOylation of YAP (Supplementary Fig. 1). Moreover, C-dots treatment in PC3 cells at 0.2 mg/ml inhibited the co-localization of YAP and SUMO in nucleus leaving the majority of YAP localized to cytosol which generally suggests that ARF and C-dots may regulate YAP through SUMOylation (Fig. 8B). In summary, our data suggest that ARF may play essential roles in non-YAP function through regulation of cytoplasm-nucleus shuttling thereby stability of YAP. Mechanically, dysregulation of phosphorylation and SUMOylation of YAP may be the insights for nuclear translocation associated with ARF. Most importantly, C-dots can stimulate ARF signaling and inhibit YAP nuclear translocation.

### Tea-derived C- dots sensitize cancer cells to rapamycin

Based on our findings that both ARF and rapamycin can inhibit nuclear YAP and C-dots can stimulate ARF, we explored whether combinatorial treatment with C-dots and rapamycin which can enhance the inhibition of YAP nuclear localization. We treated PC3 cells with differential doses of Tea C-dots at 0.1, 0.2, 0.3 mg/ml with sequential combination of rapamycin at 0.2, 0.5, 0.8 μM respectively for 4 days (Fig. 8C and D). Compared to vehicle and single treatment, the combinatorial treatment decreased cell growth more efficiently. Our data suggest that C-dots can not only be used alone to inhibit prostate cancer cell growth, but also be more efficient in inducing synergistic effect by drug combinatorial treatment with Rapamycin.

In summary, Fig. 8E indicates tea-derived C-dots may be a novel promising anti-cancer drug, and meanwhile, they can sensitize cancer cells to rapamycin. Thus, C-dots may provide a potential novel therapeutic avenue for treatment of prostate cancer.

## Discussion

In this study, we discovered that ARF dysregulates Hippo pathway through stabilizing YAP and further enhances non-nuclear YAP expression. We also found that the inhibition of YAP nuclear localization by ARF may be due to SUMOylation. The utilization of tea-derived C-Dots provides a novel avenue to enhance ARF to dysregulate YAP. Given that mTOR/YAP feedback loop is essential for YAP oncogenic function, targeting YAP by inhibition of mTOR and stimulation of ARF using the carbon dots significantly decreases PC3 cells cell growth. As PC3 cells are castration resistance type of cancer cells which most likely resistant to many types of therapy, our data demonstrate a promising avenue to treat drug resistant or relapsed cancer.

It was observed that ARF not only inhibits nuclear oncogenic YAP, but also increases total levels of YAP based on western blot analysis. Our results suggest that ARF not only stabilizes YAP, but also export YAP from nucleus. While abnormal actin stress fiber is found associated with nuclear or nuclear membrane YAP, exporting YAP from nucleus may reduce the stress fiber formation for contraction of cell during migration. In this process, ARF serves as a dual regulator for YAP, both as a tumor suppressor by enhancing YAP with reduced nuclear, and as an oncogenic factor by stabilizing total YAP to facilitate cell migration by reduced stress fiber. In addition, Pten/AKT may be a central mediator for the reversible changes of ARF function as we found ARF inhibits AKT activity. Pten/AKT are powerful in switching tumor suppressor to oncogenes^13^, it is not surprising that ARF function may be switched by AKT through the feedback loop of ARF/AKT/mTOR/YAP as we have discovered in the present work.

## Materials and Methods

### Synthesis and characterization of tea-derived C-dots

Black tea powder (Classic Assam tea) and ethylenediamine (Sigma Aldrich^®^ CAS 107-15-3) were mixed with a mass ratio of 1:4 (tea:ethylenediamine) in 60 mL of de-ionized water and placed in a Teflon lined autoclave reactor and was heated for 200 °C for 5 h. The resulting product was filtered then centrifuge at 20,000 rpm for 20 minutes to remove the bigger particles. The water in the resulting supernatant was evaporated *in vacuo* using rotavap. The C-dots were washed twice with methanol and evaporated *in vacuo* in between washings to remove the excess ethylenediamine. The remaining solvent or water in the C-dot was finally removed through heating the sample at 120 °C for 24 hours.

The morphology and particle size of the carbon dots was characterized using SmartSPM 100 scanning probe microscope (AIST-NT) in non-contact mode using high accuracy HA-NC “etalon” probes (cantilever frequency 140 kHz) at a scan rate of 1 Hz. The fluorescence excitation and emission spectra were collected at ambient temperature on a Cary Eclipse Fluorescence spectrophotometer (Agilent Technologies). Fourier transform infrared (FT-IR) spectrum was recorded on a ThermoScientific Nicolet iS5 FT-IR spectrometer using diamond attenuated total reflectance (ATR) module. Raman spectra was collected with Horiba LabRam microscope upon excitation of 633 nm using He-Ne laser (the measured power at the sample is about 1 mW).

### Cell growth assay

In the cell proliferation assay, cells were seeded in a 24-well plate at a density of 50,000 cells per well. Cells were treated with vehicle or carbon dots after 24hrs. The number of cell was determined after 4 days post treatment. In order to determine the number of cell, cells were fixed with 4% paraformaldehyde or ice-cold methanol and washed 3 times with phosphate buffer solution (PBS, pH 7.0) followed by staining with 0.3% crystal violet for 30 min. Then the images were taken using a digital camera and measured its absorbance with Evolution^TM^ 60 s UV-Visible spectrophotometer (ThermoScientific). The relative cell viability was compared with the vehicle which serves as a control.

### Immunofluorescence (IF) and confocal microscopy

The IF analysis was performed by seeding the cells on the coverslips for 24 hrs followed by the treatment with either vehicle or Tea C-dots for 3 or 24 h. Upon treatment, cells were fixed in 4% paraformaldehyde for 15 min. IF images were taken based the methods we described previously^39^, as well those described in the immunofluorescence general protocol (Cell Signaling Technology, Inc). Antibody used for F-actin was Alexa-Fluor-555-phalloidin (Molecular Probes Life technologies, 1:5000). Images were taken using Carl Zeiss Cell Observer SD confocal microscope. Primary antibodies used are: pAKT(S473) (Cell signaling), pYAP1(S127) (Cell signaling), SUMO1(Santa Cruz), SUMO2/3/4(Santa Cruz), ARF(Santa Cruz), YAP(Santa Cruz).

### ShRNA mediated knockdown of ARF gene and western blot

To knockdown the ARF gene in cell lines, stable cell lines were established as described previously^40^, alternatively, cells were transiently transfected by shRNA expression plasmid. In the western blot test, cells were harvested and lysed in buffer^39^ and subjected to SDS-PAGE (sodium dodecyl sulfate-polyacrylamide gel electrophoresis) running and immunoblotting with primary antibodies YAP (Santa Cruz), β-Actin(Sigma), β-catenin (Cell signaling), and ARF (14P02, NeoMarkers).

### Protein denaturation and turnover assay

Cells were treated with Urea at 5 mg/ml for several times. The cells were then washed with ice cold PBS twice and lysed with buffer for western blot test based on the protocol described IF and confocal microscopy section. The intensity of bands was quantified and subjected to analysis.

### Wound scratch based cell migration assay

In the scratch based wound healing assay^41^, PC3 cells were seeded in a 24-well plate and cultured until confluence. Then cells were serum-starved for 24 hrs before scratching. Wound scratching was performed using 200 μl pipette tips, and cells were gently washed twice with PBS. Pictures were taken immediately. Finally, cells were treated with regular DMEM (Dulbecco’s modified eagles medium) with 10% Fetal Bovine Serum (FBS). Wound healing rate was measured by comparison of the images using the closure distance of cells after 48 hours and at 0 hour.

### Pathway analysis of gene expression profiles

The gene expression profiles of p19^Arf^ knockout in mice as described previously^13^ were used to re-analyze ARF mediated dysregulation of signaling pathways *in vivo*. The fold changes (2.5KO vs. DKO) at least 2 of all selected genes are defined as Differentially Expressed Genes (DEGs). Fold changes (FC) above 2 is up regulation, and FC below 2 is down regulation. Then those DEGs were divided into different pathways using DAVID online tools (https://david.ncifcrf.gov/)^24,25,42,43^, and *Mus musculus* was selected as the background species.

### Statistical test

Two-tailed Student’s *t*-test was carried out for statistical analysis. The calculated value was *p* ≤ 0.05, which is regarded as statistically significant. The significance of differences in cell growth inhibition by rapamycin and combinatorial treatment were compared and calculated.
